# Situational Understanding in the Human and the Machine

**DOI:** 10.3389/fnsys.2021.786252

**Published:** 2021-12-23

**Authors:** Yan Yufik, Raj Malhotra

**Affiliations:** ^1^Virtual Structures Research, Inc., Potomac, MD, United States; ^2^United States Air Force Sensor Directorate, Dayton, OH, United States

**Keywords:** understanding, neuronal packet, active inference, complexity, cognitive effort

## Abstract

The Air Force research programs envision developing AI technologies that will ensure battlespace dominance, by radical increases in the speed of battlespace understanding and decision-making. In the last half century, advances in AI have been concentrated in the area of machine learning. Recent experimental findings and insights in systems neuroscience, the biophysics of cognition, and other disciplines provide converging results that set the stage for technologies of machine understanding and machine-augmented Situational Understanding. This paper will review some of the key ideas and results in the literature, and outline new suggestions. We define situational understanding and the distinctions between understanding and awareness, consider examples of how understanding—or lack of it—manifest in performance, and review hypotheses concerning the underlying neuronal mechanisms. Suggestions for further R&D are motivated by these hypotheses and are centered on the notions of Active Inference and Virtual Associative Networks.

## Introduction: Defining Situational Awareness and Situational Understanding

The notions of Situational Awareness and Situational Understanding figure prominently in multiple DoD documents, predicating the achievement of battlespace dominance on SA/SU superiority as, for example, in the following:

“Joint and Army commanders rely on data, information, and intelligence during operations to develop situational understanding against determined and adaptive enemies… because of limitations associated with human cognition, and because much of the information obtained in war is contradictory or false, more information will not equate to better understanding. Commanders and units must be prepared to integrate intelligence and operations to develop situational understanding” (The United States Army Functional Concept for Intelligence, 2020–2040, TRADOC 2017 Pamphlet 525- 2-, p. iii).

Distinctions between SA and SU are defined as follows:

“Situational awareness is immediate knowledge of the conditions of the operation, constrained geographically in time. More simply, it is Soldiers knowing what is currently happening around them. Situational awareness occurs in Soldiers’ minds. It is not a display or the common operating picture; it is the interpretation of displays or the current actual observation of the situation. …

Situational understanding is the product of applying analysis and judgment to relevant information to determine the relationships among the mission variables to facilitate decision making. It enables commanders to determine the implications of what is happening and forecast what may happen.” The United States Army Operations and Doctrine. Guide to FM-3-0.

**FIGURE 1:**
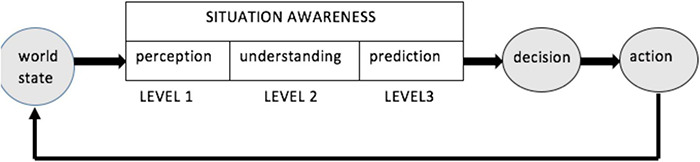
Three levels of Situation Awareness (adopted from Endsley and Connors, 2014).

Definitive publications by the originator of SA/SU concept and theory (Endsley, 1987, 1988, 1994; Endsley and Connors, 2014) identify three levels of situation awareness and associate understanding with Level 2, as shown in Figure 1.

According to the schematic in Figure 1, understanding mediates between perception and prediction. The question is: what does such mediation involve, what, exactly, does understanding contribute? The significance of such a contribution can be questioned by, for example, pointing at innumerable cases in the animal domain of going directly from perception to prediction (e.g., intercepting preys requires predators to possess mechanisms for movement prediction, as in frogs shooting their tongues to catch flying insects). The bulk of this paper is dedicated to analyzing the role and contribution of understanding in human performance, pointing, in particular, at uniquely human forms of prediction involving generation of explanations derived from attentively (deliberately, consciously) constructed situation models. Because prediction necessarily entails the consequences of action, these models must include the (counterfactual) consequences of acting. In turn, this mandates generative models of the future (i.e., with temporal depth) and implicit agency. The ensuing approach differs from that adopted in the conventional AI, as follows.

Behaviorist psychology conceptualized the brain as a “black box” and was “fanatically uninterested” in reports concerning events in the box (Solms, 2021, p.10). Borrowing from this expression, one might suggest that cognitivist psychology and AI have been “fanatically uninterested” in the role of understanding; focusing predominantly on learning and reasoning (this contention will be re-visited later in the paper). This paper argues that the capacity for understanding is the definitive feature of human intellect enabling adequate performance in novel situations when one needs to act without the benefit of prior experience or even to counteract the inertia of prior learning. The argument is presented in five parts: the remainder of part I analyzes the notions of situation awareness and situation understanding, focusing on the latter; part II outlines Virtual Associative Network (VAN) theory of understanding, part III places VAN theory in a broader context of Active Inference, part IV considers implementation (machine understanding), followed by a concluding discussion in part V. In the remainder of this part, we define some of the key notions that set the stage for and will be unpacked in the rest of the paper.

**FIGURE 2:**
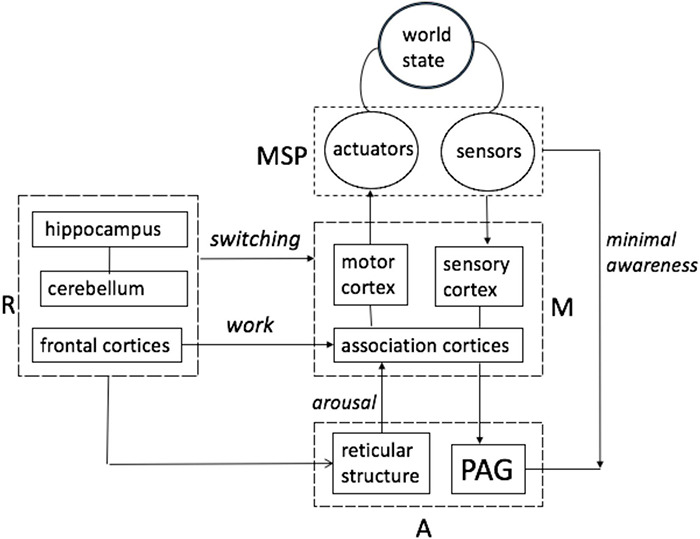
Mental models are structures formed in Memory System (M) and manipulated by Regulatory System (R). Manipulation is enabled by activation (arousal) arriving from the Activation System A (includes Reticular Activating System) and serves to organize activities in Motor-Sensory Periphery (MSP) in such a way that the resulting changes in world states are consistent with the intent originating in R.

The central tenet of this paper boils down to the notion that understanding involves self-directed construction and the manipulation of mental models. In short, planning (as inference). This idea is not original but suggestions concerning the structure of the models and the underlying neuronal mechanisms are (Yufik, 1996, 1998; Yufik and Friston, 2016; Yufik, 2018, 2021b). Figure 2 introduces some key notions in the proposal, seeking to position mechanisms of awareness and mental modeling within the brain’s functional architecture. Stated succinctly, the following treatment builds upon an understanding of the computational architecture of the only systems that evince “understanding”; namely, ourselves.

Figure 2 adopts the classical three-partite model of brain architecture in Luria (1973, 1974); Sigurdsson and Duvarci (2016), except for the inclusion of the cerebellum and Periaqueductal Grey (PAG) structure, whose role in cognitive processes—in particular the maintenance of awareness—was recently discovered (Solms, 2021). It was found that removing the bulk of cortex (in both R and M systems) while leaving the PAG intact preserves a degree of awareness (Solms, 2021). For example, hydranencephalic children (born without cortex) respond to objects placed in their hands, and surgically decorticated animals remain capable of some responses and even rudimentary learning (moreover, in some cases a casual observer might fail to notice differences in the behavior of decorticated animals and intact controls) (Oakley, 1981; Cerminara et al., 2009; Solms, 2021). By contrast, lesions of the PAG and/or reticular structure obliterate awareness (reticular structures project into cortex while PAG receives converging projections from cortex) (Solms, 2021). The architecture in Figure 2 indicates that intact PAG and RAS support *minimal awareness* (link from PAG to MSP indicates awareness achieved in the absence of the cortex) while an interplay of all the other functional systems produces a hierarchy of awareness levels above the minimal.

### Levels of Awareness

To define levels of awareness, one needs to conceptualize the world as generating a stream of stimuli and cognition as a process of assimilating sensory streams aimed to extract energy and sustain energy inflows (these crucially important notions will be re-iterated throughput the paper). With these notions in mind, the following levels of awareness can be identified.

1. *Minimal awareness* (*“vegetative wakefulness,”* the term is due to Solms, 2021, p. 134). Streams of sensory stimuli are experienced as flux (noise).
2. *Selective awareness.* Organism responds to fixed combinations of contiguous stimuli as they appear in the flux (as in frogs catching flies).
3. *Discriminating awareness.* “Blobs” with fuzzy boundaries emerge in perceptual synthesis comprising some contiguous stimuli groupings with varying correlation strength inside the groups.
4. *Differentiating awareness.* Different stimuli compositions are assimilated into “blobs” that are sharply bounded and segregated from the surrounds (“blobs” subsequently turn into distinct “objects,” as in telling letters apart).
5. *Recognition-based awareness.* Variations in stimuli compositions in the objects are differentiated (stated differently, different stimuli compositions are experienced as manifestations of the same object, as in recognizing letters in different fonts or handwriting).
6. *Context-based awareness.* The perceptual recognition of objects is influenced by their surrounds (think of the often-cited example of perceiving a shape that looks like a distorted letter A or distorted letter H, depending on its appearance in the middle of C_T or the beginning of _AT).
7. *Understanding-based awareness.* This level is qualitatively different from the preceding levels: all levels deal with learning, i.e., developing memory structures reflecting the statistics of correlation, contingencies and contiguity in the world. By contrast, this level produces and manipulates complex relational structures (mental models) uprooted from such statistics (accounting for non-contiguous and weakly correlated, sparse stimuli groupings) — in other words, compositions and counterfactuals. To illustrate the distinction: the statistics of English texts would suffice for resolving the “_AT or C_T” ambiguity but not for understanding the expression “hats on cats” (when was the last time you saw or read about cats wearing hats?).

Arguably, Figure 1 refers primarily to understanding-based situation awareness. It is informative to note that cells in prefrontal cortices represent the association of sensory items of more than one sensory modality, integrate these items across time and participate in performing tasks requiring reasoning and manipulation of complex relational structures (Kroger et al., 2002). Construction and manipulation of complex relational structures underlies understanding. More precisely, understanding enables construction of models expressing unlikely correlations (like cats in hats), while sometimes failing to register some precise and routinely encountered ones (e.g., medieval medicine for centuries failed to see the relation between a beating heart and blood circulation, placing the source of circulation in the liver). This paper offers ideas seeking to account for both the strengths and the weaknesses of the understanding capacity. Three pivotal notions (*work, switching, and arousal*) are referenced in Figure 2 (labeled in italics).

#### Work (Mental Work)

Operations on mental models demand effort and energy, in the same manner as are those demanded by any bodily (i.e., thermodynamic) work, such as running or lifting weights.

#### Switching

The functional architecture in Figure 2 is shared across many species, except for the capability to temporarily decouple mental models from the motor-sensory periphery and environmental feedback. The emergence of this regulatory capacity—to allow such decoupling— underwrites the development of an understanding capacity that is uniquely human and enables uniquely human skills, such as extending the horizon of prediction reach from the immediate to an indefinitely remote future and extending actions reach from objects in the immediate surrounds to indefinitely distant ones, etc.

#### Arousal

Regulation of arousal (energy distribution in the cortices) is an integral and critical ingredient of mental modeling. In particular, modeling is contingent on maintaining the stability and integrity of neuronal structures in the cortices implementing the models. Resisting entropic erosion and disintegration of the structures require sustained inflows of metabolic energy. These ideas will be given precise definitions that will be mapped onto a simple mathematical formalism.

To summarize, three different brain mechanisms have been identified: those that circumvent the cortex, those that engage the cortex, and those mechanisms that are realized in the cortex and are temporarily disengaged from the motor-sensory periphery (*switching).* The former two mechanisms underlie learning and are shared among multiple species, including humans, while the latter is unique to humans and underlies understanding. The proposal so far is derived from the following conceptualization: (a) the world is a process or stream (not a “static pond”), (b) cognition is a process of adapting an organism’s state and behavior to variations in the stream, and (c) the adaptation are powered by energy (*work*) extracted from the stream and distributed inside the system (*regulation of arousal*). Understanding complements learning: learning extrapolates from past experiences, while understanding overcomes the inertia of learning when encountering new conditions with no precedents. Overcoming inertia is an effortful process that can fail but provides unique performance advantages when it succeeds. It was noted that AI and the cognitivist school of thought have downplayed the role of understanding in performance.

The concept of Situation Awareness in Figure 1 predicates awareness on understanding, consistent with the notion of understanding-based awareness introduced in this section (note that Figure 1 does not address learning. Accordingly, this article does not expand on relations between learning and understanding, except for the comments in the preceding paragraph). The next section takes a closer look at the process of understanding and provides examples of its successes and failures.

### Situational Understanding

Colloquially, “understanding” denotes an ability to figure out what to do when there is no recipe available and no precedent or aid to consult. The dictionary formulation captures the essence of that ability defining understanding (comprehension, grasp) as “apprehending general relations in a multitude of particulars” (Webster’s Collegiate Dictionary). In science, relations are expressed by equations. Accordingly, in understanding scientific theory T, apprehending general relations takes the form of “recognizing qualitatively characteristic consequences of T without performing exact calculations” (Criterion for Intelligibility of Theories) (de Regt, 2017, p. 102). The experience of attaining scientific understanding was described by Richard Feynman as having

“some feel for the character of the solution in different circumstances. … if we have a way of knowing what should happen in given circumstances without actually solving the equations, then we “understand” the equation, as applied to the circumstances. A physical understanding is a completely unmathematical, imprecise, and inexact thing, but absolutely necessary for a physicist (Feynman, c/f de Regt, 2017, p. 102).

The Criterion subsumes epistemic and pragmatic aspects of theoretical understanding, i.e., producing explanations of various phenomena and applications in various situations. Figuratively, understanding cuts through the “fog of war” (Clausewitz, 2015/1835) when apprehending battlefield situations and the “fog of mathematics” when apprehending scientific theories.

These notions are consistent with conceptualizations of understanding in psychology [theory of understanding (Piaget, 1975, 1978), theory of fluid and crystallized intelligence (Cattell, 1971, 1978)], emphasizing ability to apprehend relations under novel conditions and in the absence of practice or instruction [fluid intelligence (Cattell, 1971, 1978)]. The term “situational understanding” connotes changing conditions, with situations transforming fluidly into each other (e.g., attack- halt - withdraw - attack…, etc.). The remainder of this section presents examples of situational understanding, prefaced by a brief analysis (anatomy of the process) in the next two paragraphs. These examples are followed by preliminary suggestions regarding the underlying mechanisms.

Reduce a multitude of objects to just two, A and B, and consider situation “A moves towards B.” In reaching decision that A attacks B, three stages can be identified, with the first one being readily apparent, while the significance of the second is easily overlooked. First, one must perceive A and B, which involves distinct activities (alternating attention between A and B) producing two distinct memory elements (percepts). Second, percept A and percept B must be juxtaposed (grouped), i.e., brought together and held together in memory (call it “working memory”). The task appears to be easy when the activities follow in tight succession (e.g., both A and B are within the field of view) but not so easy when they are separated by large time intervals. The third stage involves establishing a relation, which is predicated on the success of the preceding stages. The second stage is crucial: arguably, the development of understanding was launched by the emergence of mechanisms in the brain allowing juxtaposition of percepts separated by large stretches of time. At this point, it is informative to note that a recent theory concerning the origins of language capacity in the humans associated this capacity with the emergent availability of mental operation (called Merge) where disjoint units A and B are brought together to produce a new unit (A B) → C amenable to subsequent Merge, (C Q) → Z, and so on (Chomsky, 2007; Berwick and Chomsky, 2016).

Identifying stages in the understanding process helps to appreciate the staggering challenges it faces. When experiencing A, how does the idea of relating A to B come to mind? Figuring out this relation takes effort but the very expression “coming to mind” connotes spontaneity. Accordingly, understanding can break down if the effort is insufficient and/or spontaneous mechanisms fail to deliver. The point is that understanding involves dynamic interplay of deliberate operations and automatic memory processes triggered by the operations that might or might not converge in a grasp. To exemplify the point, consider a syllogism (say, “all humans are mortal, Socrates is a human, therefore Socrates is mortal”). It might appear that the conclusion inescapably follows from the premises but that’s an illusion: one might be aware of each of the premises individually but fail to bring them together, and/or the conclusion might either not come to mind or get suppressed upon arrival. Some extreme examples of failed and successful situational understanding are listed next.

On May 17, 1987, the USS Stark on patrol in the Persian Gulf was struck by two Exocet AM-39 cruise missiles fired from an Iraqi F-1 Mirage fighter. An investigation revealed that the aircraft was detected by AWAC (Airborne Warning and Control) patrolling in the area and identified as “friendly.” Due to the erroneous initial identification, the captain and crewmembers on the frigate ignored subsequent aircraft maneuvers that were unambiguously hostile (turning, descending and accelerating in the direction of the ship) which resulted in a loss of 37 lives and severe damages to the ship (Miller and Shattuck, 2004).

Between May 9th and June 14th in 1940, France was invaded by the German army. France was one of the major military powers in Europe that maintained adequately equipped forces and, besides, invested tremendous resources in erecting state-of-the-art fortifications on its northern border (the Maginot Line). Despite these preparations, France suffered a historic defeat. Massive literature has been produced over many decades, analyzing the course of events and suggesting various reasons for this colossal and catastrophic failure. A book published in 1941 by a competent French author (served as a liaison officer in the British army during WWI) summarized discussions with French and British officers and political figures before and after the events in question, His analysis offers what appears to be a plausible account and explanations (Maurois, 1941). In particular, the book pointed out that French military and political authorities overestimated the efficiency of the Maginot defenses which stemmed, interestingly, from French technical advances and a sense of engineering superiority. French generals determined Maginot fortifications to be impenetrable on the grounds that they “can be built so rapidly that, in the time necessary for an enemy to take a first line, the defending army can construct a second …” (Maurois, 1941, p. 42). A full range of state-of-the-art technologies (reconnaissance photography, advanced communications, etc.) was employed, the terrain was meticulously examined and mapped out and artillery ranges were calculated in advance. “These painstaking labors assured absolute precision of fire. The spotters in front of the forts had before them photographs of the country divided into numbered squares. Perceiving the enemy in square 248-B, all they would have to do was murmur “248-B” into the telephone, and 10 s later the occupied zone would have been deluged with shells and bullets” (Maurois, 1941, p. 48). In short, a confident consensus was predicting that the Maginot fortifications will never be broken through. These predictions turned out to be correct: Germans went around and bypassed the Maginot Line entirely, invading Paris on June 14, 1940.

On January 15th, 2009, the Airbus A320-214 flying from LaGuardia Airport in New York struck a flock of geese during its initial climb out. The plane lost engine power, and ditched in the Hudson River off midtown Manhattan just 6 min after the take off. The pilot in command was Captain Sullenberger (CS), the first officer was Skiles. The bird strike occurred 3 min into the flight and resulted in an immediate and complete loss of thrust from both engines. At that instant, Skiles began going through the three-page emergency procedures checklist and CS took over the controls. In about 30 s, he requested permission for an emergency landing in a nearby airport in New Jersey (NJ) but decided on a different course of action after the permission was granted. Having informed controllers on the ground about his reasons (“We can’t do it”) and intents (“We’re gonna be in the Hudson”), CS proceeded to glide along and then ditch the aircraft in the river. All the 155 people on board survived against a staggeringly bad odds (Suhir, 2019).

The underlying mental process in all three incidents involves item grouping, success or failure in the overall task performance depended on how that step was accomplished. One more example will help to illustrate this contention. Analysis of eye tracking records of ATC controllers revealed latent grouping of aircraft signatures on ATC displays which appeared to be motivated by gestalt criteria (e.g., relative proximity). The probability of detecting possible collision was higher for the aircraft residing in the same group (A B) than for those residing in different groups, (A B) (C D). It was hypothesized that novice controllers could not disable gestalt grouping but the more skilled ones developed a capacity for overcoming its impact on performance (Landry et al., 2001; Yufik and Sheridan, 2002). We now turn to analyzing these examples.

In the USS Stark incident, three items had to be accounted for in the Captain’s decision process: *A* = own ship, *B* = AWAC, and *C* = F1 Mirage. In the Captain’s mental model, grouping (A B) was the dominant one (i.e., attributing significance to any item C respective A relied entirely on B). The “friendly” determination rendered C irrelevant to A and removed it from consideration. Hence, the “blind spot” on the Iraqi F-1 Mirage fighter whose behavior was displaying signs of attack that could not be any clearer: the aircraft was ascending away from the ship but then sharply changed its course and started descending and accelerating toward the ship.

French military planners recognized the possibilities of German bypassing maneuvers (e.g., attacking through Belgium) but “rationalized them away,” i.e., worked out lines of reasoning that rendered them highly unlikely and, ultimately, have forced them out of consideration. French strategic thinking was structured by the experience of trench warfare in WWI when opponents were facing each other from fortified positions and conducted frontal assaults to break through each other’s defenses. As a result, the mental models of the leading strategists were focused on the fortifications and defended areas in front of them (A B) while turning a “blind eye” to the adjacent areas (C). Because of the influence earned by the generals in their past victories, these models became the dominant view across the French military, intelligence and political communities. Common sense would suggest that the Maginot Line needed to be “prolonged along the Belgian frontier by fortifications that were perhaps less strong but nevertheless formidable. I received one of the greatest shocks of my life when I saw the pathetic line…which was all that separated us from invasion and defeat” (Maurois, 1941, p. 19). The point is that experience- sculpted models can produce pathological tunnel vision which cannot be remedied by reasoning – to the contrary, reasoning confined to the same tunnels can only make them more rigid. Practical validation, an otherwise uncompromisingly reliable criteria, could also do a disservice (one can imagine placing targets in front of the fortifications and, after some extra practice, having them destroyed, not in 10 but in 8 s).

The Airbus A320-214 incident prompted a thorough investigation and analysis that engaged the most advanced investigative and analytic tools available (Suhir, 2012, 2018, 2019; Suhir et al., 2021). Unlike in the previously cited scenarios, this analysis had unlimited access to complete records and could use computer modeling and testing in flight simulators to validate the conclusion. The analysis was centered on probabilistic risk estimates accounting for the human error stemming from imbalances between human capacities (Human Capacity Factor, or HCF) and mental workload (MWL). Ten major contributors into HCF were identified: (1) psychological suitability for the given task, (2) professional qualifications and experience, (3) level, quality, and timeliness of past and recent training, (4) mature (realistic) and independent thinking, (5) performance sustainability (predictability, consistency), (6) ability to concentrate and act in cold blood (“cool demeanor”) in hazardous and even in life threatening situations, (7) ability to anticipate (“expecting the unexpected”), (8) ability to operate effectively under pressure, (9) self-control in hazardous situations, and (10) ability to make a substantiated decision in a short period of time. Captain Sullenberger was expected to score high on the majority of these factors. In simulator tests, four pilots were briefed in advance about the entire scenario in full detail and then exposed to simulated conditions immediately after the bird strike. Knowing in advance what to expect, all four were able to land the aircraft. However, when a 30 sec delay was imposed (the time it took Sullenberger to assess the situation and decide on the course of action), all four pilots crashed (Suhir, 2013).

**FIGURE 3:**
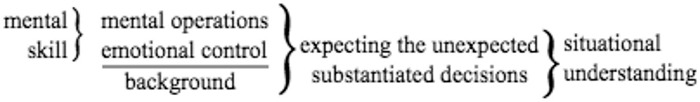
Situational Understanding is a product of background (knowledge, training) and mental skills. A solid horizontal line underscores that skills operate on top of the background.

Applying the HCF metric to other examples, it can be suggested that HCF scores reflect capacity for situational understanding, ranging from the bottom low to exceptionally high. For the purposes of this paper, the ten factors can be divided into four groups three of which can be roughly mapped onto components in the architecture in Figure 2 (roughly, factors 2 and 3 relate to Memory, factors 1, 5, 6 relate to Activation and factor 4 and 9 relate to Regulation) while the forth group is made up of 7, the ability to anticipate (“expecting the unexpected”), and 10, the ability to make a substantiated decision in a short period of time) relate to Situational Understanding, conceptualized here as a product of interplay between the other three groups. Figure 3 re-phrases this suggestion.

Mental skills operate on top of background, including knowledge and skills acquired in training, but are qualitatively different from those. The distinction extends from responding to unexpected eventualities to constructing scientific proofs or theories where the process of selecting and applying the rules of the theory at each stage cannot be itself governed by another set of rules (de Regt, 2017). In the Airbus incident, emergency rules and training dictated either consulting the emergency checklists or seeking possibilities for heading to the nearest airport. Following either of these courses of action would be both rational (not random or unreasonable) and in line with the cumulative experience in the aviation community, but would have surely killed all on board.

There are five points to be made here. First, an NJ landing was initially considered by CS and implicitly supported by controllers on the ground, as evidenced by the granting landing permission. Second, CS could not even start analyzing the NJ option (i.e., considering the current altitude, airspeed, distance, wind, aircraft characteristics, etc.) but could only develop a “feel” that it would not work out. Third, despite the absence of analysis, the “feel” allowed a substantive decision (“We can’t do it”). Forth, having developed the “feel,” CS acted on it resolutely, entailing another substantive decision (“We’re gonna be in the Hudson”). Fifth, CS did not know the future but performed comparably to or better than pilots who knew the scenario in advance. In short, CS understood the situation in a process involving three distinct mental operations, as follows.

1. Forceful re-grouping, not derived from any rule or precedent (*jump*, ↱).
  (*AB*)↱(*AC*), here A is the aircraft, B is the New Jersey airport and C is the Hudson River.
  The expression reads as “group (A B) *is jumped* to group (A C).”
2. Alternating attention between members inside a group while envisioning variations in their characteristics (*coordination*, ⇌).
  [*var* (A) ⇌*var* (C)], attention alternates between envisioning variations in the aircraft behavior [*var* (A) (e.g., changes in attack angle) and changes along the riverbed (*var* (C)] (e.g., changes in width, curvature, etc.). Reads as “A *is coordinated* with C.”
3. Forcefully iterating *coordination* until a particular coordination pattern (*relation)* is apprehended (*blending*, ↭).
  [(*var* (A) ↭*var* (C)], reads as “A *is blended* with C.” *Blending* transforms a coordinated group into a cohesive and coherent functional whole so that, e.g., envisioning variations in one member *brings to mind* the corresponding variations in the other one (thinking of ditching near a particular spot brings to mind the required changes in aircraft behavior and, vice versa, envisioning changes in the behavior brings to mind the corresponding changes in the location of the spot). *Blending* establishes *relation R* on the group [*var* (A) ⇌*var* (C)] → (A *R* C) which gets expressed in substantive decisions (“We’re gonna be in the Hudson”) and gives rise to probability estimates for coordinated activities (“chances of a successful ditching are not too bad”) and their outcomes (more on that in the next section).

Operations *jump*, *coordination*, and *blending* participate in the construction of mental models, culminating in *blending* which makes one *aware of*, i.e., anticipate direct and indirect results of one’s actions without considering situational details. Intuitive appreciation of this dualistic relationship between awareness and understanding seems to be the motivation in the Situational Awareness concept and the SA schema in Figure 1.

To summarize, understanding involves the construction of mental models that make an adequate performance possible when exploring unknown phenomena and/or dealing with unforeseeable eventualities in the otherwise familiar tasks. In the latter case, understanding enables decision processes that are substantive, short (as compared to the duration of the task), rely on minimal information intake, and achieve results approximating those one would achieve had all the eventualities been known in advance. Importantly, mental models not only generate likelihood estimates for future conditions but make one envision them and then actively regulate motor-sensory activities consistent with the anticipated conditions and in coordination with motor-sensory feedback (hence, the “expecting of the unexpected”). Note that simply to decide or choose immediately requires there to be a space of policies or narratives to select from. The position offered in this paper is that this necessarily entails the ability to represent the (counterfactual) consequences of two or more courses of action—and to select optimally among these representations. What brain mechanisms could underlie this capability?

## The Virtual Associative Network Theory of Mental Modeling

The VAN model was motivated by one paramount question (“How does understanding work?”) and stems from the three already familiar ideas that can be re-stated as follows:

(1) The world is a stream, and brain processes are dynamically orchestrated to adapt organism’s behavior to variations in the stream.
(2) The brain is a physical system, wherein all processes need to be powered by energy extracted from the stream.
(3) Physical systems are dissipative, so any re-organization takes time (instantaneous reorganization would require infinite energy). As a result, adaptive re-organizations are necessarily anticipatory.

Taken together, these ideas entailed the following two hypotheses:

(a) the evolution of biological intelligence has been (selectively) pressured to stabilize energy supplies above some life-sustaining thresholds and
(b) human intelligence was brought about by biophysical processes—discovered by evolution—that allowed for two fundamental mechanisms to emerge: mechanisms that stabilized energy supplies from the outside and those minimized dissipative losses and energy consumption inside the brain. These mechanisms culminate in the uniquely human capacity for understanding, as outlined in the remainder of this section. The next section will suggest a hand-in-glove relationship between thermodynamic efficiency and variational free energy minimization (VFEM) (Friston, 2009, 2010).

Note that the VAN approach is orthogonal to that expressed in the perceptron (neural nets) idea: dynamically orchestrated neuronal structures vs. fixed structures (after the weights are settled), input streams where stimuli combinations are never twice the same vs. recurring inputs. Crucially, accounting for energy and time is integral to the VAN model and alien to the perceptron framework. In short, the VAN and perceptron models reside in different conceptual terrains. The appeal of the former is the possibility of quickly reaching a point where a theory of understanding can be articulated. Technically, the distinction between perceptron and related reinforcement learning and VAN is the distinction between an appeal to the Bellman optimality principle (any part of an optimal path between two configurations of a dynamical system is itself optimal) and a more generic principle of least action where action corresponds to energy times time. VAN and the free energy principle (a.k.a. active inference) share exactly the same commitments. Note that formulating optimal behavior in terms of a principle of least action necessarily involves time—and the consequences of behavior.

To set the stage, return to Figure 2, and think of the world as a succession or stream of states *S*_*i*_,*S*_*j*_… arriving with time interval τ_1_,and think of the brain as a pool of N binary neurons. Interaction is driven by the need to extract energy from the world in the amounts sufficient for the pool’s survival. Anthropomorphically, this entails recurring cycles of inquiring (What is the current state of affairs in the world?) and forming responses (What shall I do about it?). The sequence of “inquiries” at each cycle can be expanded: What is the state? What can I do about it? What shall I do about? How shall I do it? and so on. Also, different types of neurons can be envisioned and mapped onto different components in the architecture in Figure 2 (sensory neurons, motor neurons, etc.).

Whatever the composition of the pool and the content and order of the inquiries, activities in the pool boil down to selectively flipping (exciting or inhibiting) neurons in a particular order. Make two assumptions: (a) each state *S*_*i*_can emit energy reward _*i*_ ranging from 0 to some maximum ${△}_{i}^{max}$, depending on the order and composition of “flippings” in the pool and (b) each “flip” consumes energy d (at the first approximation, let all flips be powered by the same energy amount). The problem facing the pool can be defined now as maximizing energy inflows while minimizing the number of flips. It will be argued, in four steps, that understanding involves a particular strategy for satisfying this dual objective (step 4 defines architecture for understanding).

### Step 1. Neuronal Groupings

A pool of N binary neurons admits 2^*N*^ configurations so that, in principle, selecting a rewarding configuration for a particular world state can pose a problem that grows in complexity with the size of the pool (associating complexity measure with the number of options). The problem is alleviated when choices are dictated by the world state itself (i.e., each stimulus in the composition of *S*_*i*_excites particular neurons) but, otherwise, the pool needs to choose between 2^*N*^ options.

**FIGURE 4:**
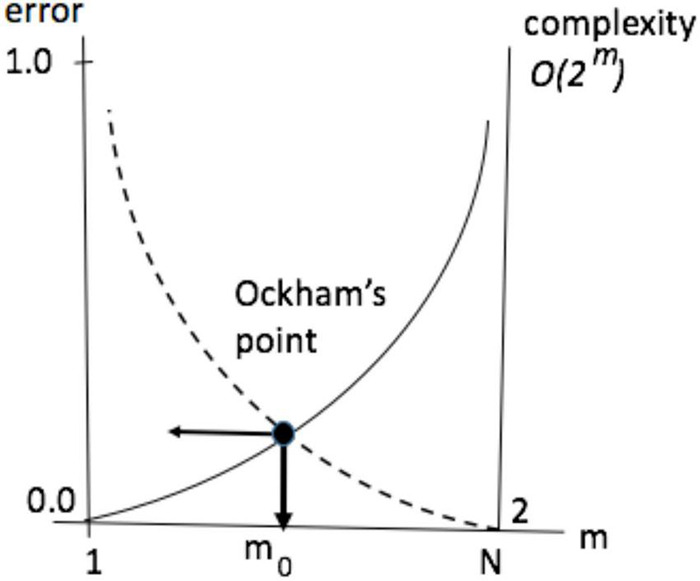
Self-partitioning in the neuronal pool radically impacts pool’s capacities in responding to world streams and involves trade-offs between time and accuracy, as a function of group size. The relationship is non-linear, creating long tail areas where, on the one side, sacrificing speed (increasing the number of groups) produces no appreciable improvements in accuracy (“useless details”) and, on the other side, small speed gains produce quickly increasing errors (“useless generalities”). A narrow inflection zone (Ockham’s point, or *O*-point) lies between the tail areas.

Assume that a mechanism exists to partition the pool into *m* groupings [call them neuronal assemblies (Hebb, 1949, 1980)] such that all the neurons in every group behave in unison. Such partitioning would offer more efficiency, reducing the number of choices from 2^*N*^ to 2^*m*^. The remedy is radical because it not only puts a lid on complexity growth but causes complexity to decrease steeply with the size of the pool (e.g., partitioning pool of 10 neurons into 5 groups yields 2^5^: 1 reduction in the number of options while having 5 groups in a pool of 100 neurons obtains 2^95^: 1 reduction). Complexity reduction translates into an increase decision speedup (e.g., equating complexity to time-complexity, by assuming one choice per unit time) and internal energy savings. Indeed, complexity reduction can be regarded as underlying all (i.e., universal) computation; in the sense of algorithmic complexity and Solomonov induction. The benefits of compression and complexity minimization come at a price: imploding complexity is accompanied by exploding error — as the loss of degrees of freedom precludes an accurate prediction. This trade-off between accuracy and complexity is illustrated in the notional diagram in Figure 4 (error η_*i*_ is measured by the difference between $\mathrm{energy}\mathrm{gain}{△}_{i}^{N}$ obtainable in the pool without partitioning and $\text{gain}{△}_{i}^{m}$ yielded by *m*- partitioning).

To illustrate, veering to the left of the *O*-point in the Airbus accident would be akin to CS receiving advice “aviate, navigate, communicate” from the ground controllers, which is a paramount principle in aviation human factors (Wiener and Nagler, 1988) but hardly a useful guidance under the circumstances, while veering to the right would be like offering a refreshment course in plane aerodynamics. Depending on the task, the relative width of Ockham’s zone on the group size axis can be very small so the ability to stay within it (e.g., not going through emergency checklists, discontinuing communications, etc.) can make vital differences in the performance outcomes. Put simply, there is a right level of “grouping” or “course graining” that provides the right balance between accuracy and complexity. Statistically speaking, this corresponds to maximizing marginal likelihood or model evidence.

Arguably, the emergence of grouping mechanisms in the neuronal substrate was a major discovery in the evolution of biological intelligence (from sensing to understanding). Accordingly, the concept of neuronal assembly remains the single, most revealing idea at the foundation of neuroscience (Hebb, 1949, 1980). Neuronal groupingopened new avenues for development, *via* fine-tuning and manipulation of the groups. Pursuing such adaptive improvements equates to bending curves and “pushing” the Ockham’s point toward obtaining minimal error in the smallest number of groups (see Figure 3). It was subsequently argued that thermodynamics has been doing the “pushing” (Yufik, 1998, 2013), we will touch on that later.

### Step 2. Varying and Coordinating Group Activities

**FIGURE 5:**
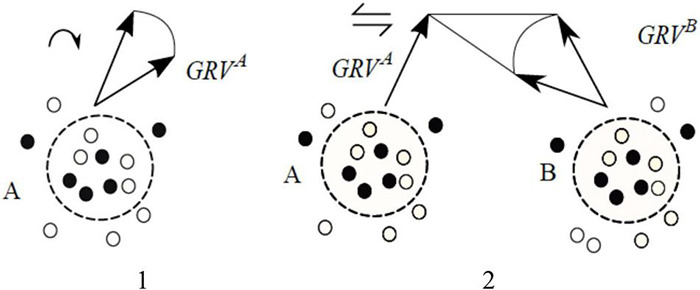
**(1)** Patterns of excitation-inhibition within groups can be varied, which can be expressed as rotation of group response vectors (symbol ↷ denotes operation “rotation of group response vector”). **(2)** Movements of group response vectors can be coordinated: every position of *GRV^A^* determines a range of admissible positions for *GRV^B^*, and vice versa, movement of one vector causes re-positioning of the other one (i.e., thinking of changes in A *brings to mind* the corresponding changes in B).

On-off decisions on neuronal groups can be dynamically nuanced to allow more close tracking of the world stream, by, first, tuning receptive fields in individual neurons and, second, by varying excitation–inhibition balance within each. A convenient expression of that strategy can be obtained by summing up response vectors of all the participating neurons in a group to obtain “group response vector” (GRV) and then characterizing activity variations inside a group as patterns in the movement of GRV. Finally, mechanisms for inter-group coordination would develop on top of the mechanisms for controlling intra-group variations. Coordination involves mutual constraints, i.e., variations in one group can both trigger and limit the range of variations in another one. Mutual constraints reduce the number of options, thus shifting the *O*-point down and to the left. Figure 5 depicts progression from intra-group variation to inter-group coordination.

One of the cornerstone findings in neuroscience revealed that movement control (e.g., extending hand toward a target) involves a rotation of response vectors in groups of motor neurons, as in Figure 5.1 (Georgopoulos and Massey, 1987; Georgopoulos et al., 1988, 1989, 1993). Accordingly, complex coordinated movements can involve coordinated rotation of group response vectors in synergistic structures in the motor cortex comprising multiple neuronal groups (Latash, 2008).

### Step 3. Neuronal Packets and Brain Energy Landscapes

**FIGURE 6:**
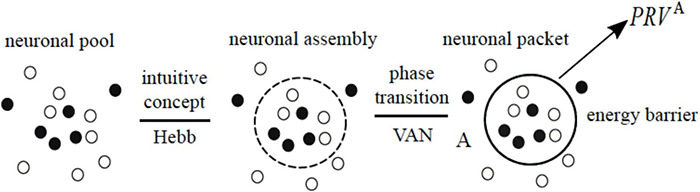
The idea of assembly expresses the notion that groups of tightly associated neurons form cohesive units distinct from their surrounds in the network (associative links are not shown). The notion of a neuronal packet expresses, in the most general terms, a mechanism for forming and stabilizing such units in a material substrate (i.e., phase transition and emergence of an energy barrier in the interface between the phases). *PRV^A^* denotes “packet response vector.”

The following hypotheses is central in the VAN model: neuronal assemblies are formed as a result of phase transitions (Kozma et al., 2005; Berry et al., 2018) in associative networks, when tightly associated subnets become separated by energy barriers from their surrounds (c.f., the formation of droplets in oversaturated vapors). The term “neuronal packet” was coined in Yufik (1998) to denote neuronal assemblies bounded by energy barriers. It can be argued that Hebb’s insight recognizing assemblies as functional units in the nervous system (as opposed to attributing this role to individual neurons) necessarily implied the existence of biophysical mechanisms that keep such assemblies together, separate them from the surrounding network and make it possible to manipulate them without violating their integrity and separation. On that argument, the VAN theory only makes explicit what was already implied in the idea of neuronal assembly. Figure 6 elaborates this contention.

**FIGURE 7:**
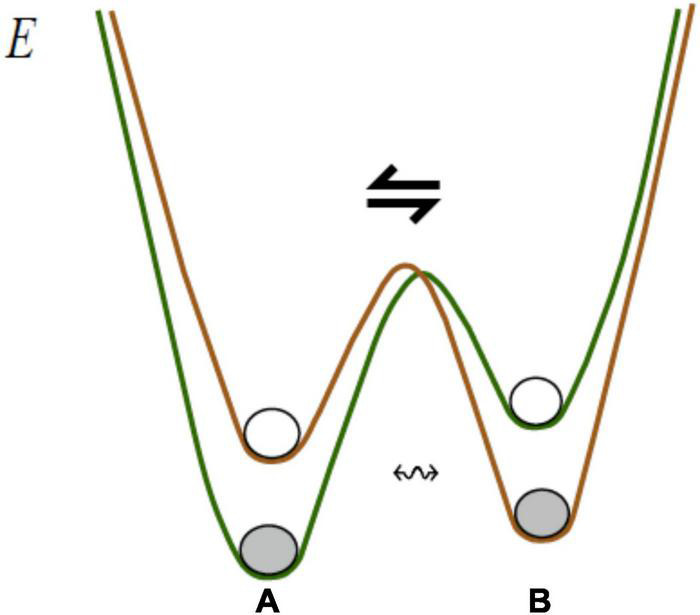
Associative structures reside in continuous energy landscape. Coordinating objects A and B occupying different minima (A B) → (A ⇋B) requires repetitive climbing over the energy “hill” between the minima. Deformation in the landscape (lowering the “hill”) enables *blending* (A ⇋B) → (A ↭B), producing a structure where A and B remain distinct and, at the same time, capable of constraining each other’s behavior.

Associating boundary energy barriers with biological neuronal groups expresses a non-negotiable mandate that operations on such groups, including accessing the neurons inside, varying excitation-inhibition patterns in the groups, removing neurons from a group, etc. all involve work and thus require a focused energy supply to the group’s vicinity sufficient for performing that work. Multiple packets establish an energy landscape in the associative network, as shown in Figure 7.

Packets are internally cohesive and externally weakly coupled (i.e., neurons in a packet are strongly connected with each other and weakly connected with the neurons in other packets), the cohesion/coupling ratio in a packet determines the depth of energy “well” in which it resides: the deeper the well, the more stable the packet, which translates into reduced amounts of processing and higher degree of subjective confidence when packet contents are matched against the stream [packets respond to correlated stimuli groupings, the number of matches sufficient for confidently identifying the current input decreases as the cohesion/coupling ratio increase (Malhotra and Yufik, 1999)]. Changes in the landscape, as in Figure 6, result from changes in arousal accompanying changes in subjective values (importance) attributed to the input (objects, situation): the higher the value attribute to an object, the deeper the corresponding well becomes (more on that shortly).

The notions of neuronal packets and energy landscape in Yufik (1996, 1998); Yufik and Sheridan (2002) anticipated experimental and theoretical investigations of cortical energy landscapes (Watanabe et al., 2014; Gu et al., 2017, 2018; Kang et al., 2019). However, packet energy barriers are amenable to direct experience, as was first intimated by William James in his classic “The Principles of Psychology” back in 1890, as follows. To access an item in memory, one must make attention

“linger over those which seem pertinent, and ignore the rest. Through this hovering of the attention in the neighborhood of the desired object, the accumulation of associates become so great that the combined tensions of their neural processes break through the bar, and the nervous wave pours into the track which has so long been awaiting its advent” (James, 1950/1890, v. 1, p. 586).

**FIGURE 8:**
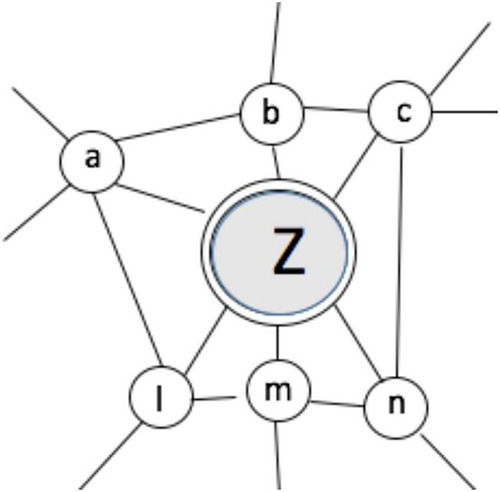
Accessing contents of packet Z requires sustained attention in the associative neighborhood until effort is mounted sufficient for overcoming “resistance”(i.e., boundary energy barrier) (adopted from James, 1950/1890, v. 1, p. 586).

To appreciate the insightful metaphor “breaking through the bar,” think of desperately trying to recollect the name of an acquaintance that escaped you just at the moment you were making an introduction. With a stunning insight and vividness (Figure 8), James describes the experience of mounting mental effort to access packet’s internals from the surrounding associative structure:

“Call the forgotten thing Z, the first facts with which we felt it was related, a, b, c and the details finally operative in calling it up, l, m and l. …The activity in Z will at first be a mere tension, but as the activities in a, b and c little by little irradiate into l, m, n, and as all these processes are somehow connected with Z, their combined irradiation upon Z …succeed in helping the tension there to overcome the resistance, and in rousing Z to full activity” (James, 1950/1890, v. 1, p. 586).

Building on the notions in Figure 7, assume, first, that Z admits a number of distinguishable states *Z* = *Z*_1_, *Z*_2_, …, *Z_k_*, second, another packet *Q* = *Q*_1_, *Q*_2_, …., *Q_m_* exists somewhere in the associative network, third, attention alternates between varying states in Z *Z*_*1*_ → *Z*_2_ → …→ *Z_k_* and Q *Q*_1_→ *Q*_2_→…. →*Q*_*m*_ (i.e., rotating packet vectors) until, finally, a particular form of coordination between the variation patterns is established (relation r), producing a coordinated relational structure Z *r* Q. With that, a model is formed expressing variations in the world stream in terms of objects, their behavior and inter-object relations (more on that shortly). Transporting James’ vivid account into modern context, “hovering of the attention” can be compared to burning fuel in a helicopter hovering over a particular spot, and inter-packet coordination is like keeping two helicopters airborne and executing different but coordinated flight patterns. Finally, forcing changes in the landscape and establishing coordination, as in Figure 6, is analogous to letting the helicopters roll on the ground and having them connected by a rod to coordinate their moves. The following two suggestions reiterate these notions more precisely.

First, alternating between the packets is an effortful process critically dependent on the strength of “resistance” offered by the energy barrier: excessive height will make the packets mutually inaccessible while low barriers will make them less stable and thus disallow sustained and reproducible variations. In short, the process is contingent on maintaining a near-optimal height of energy barriers throughout the landscape, as suggested in Figure 6.

Second, establishing relations replaces effortful alternations between packets with effortless (automatic) “facilitation” (the term is due to Hebb, 1949). Stated differently, a rule “varied together, coordinated together” can be suggested as a complement to Hebb’s “fire together, wire together” rule, extending its application from neurons to packets. Facilitation underlies the experience of *coming to mind* when thinking of changes in Z *brings to mind* the corresponding changes in Q, as in Figure 5.2. More generally, packets become organized (blended) into a model yielding the capacity to “have some feel for the character of the solution ….without actually solving the equations” (Feynman, see section “The Virtual Associative Network Theory of Mental Modeling”). Stated differently, one becomes aware of the direction in which changes in one model component impact behavior of the other ones and of the entire composition, consistent with the insight expressed in Figure 1. Situational “feeling” is coextensive with reaching understanding and obtaining complexity reduction in the modeling process on a scale ranging from small in simple situations to astronomical in complex ones.

To appreciate the significance of the benefit, think of a most rudimentary task, e.g., a chimpanzee connecting sticks and climbing on top of piled boxes to reach some fruit. Connecting sticks involves trying out different random variations until the proper coordination is encountered (Koehler, 1999). Connected sticks become a physical unit that can be physically coordinated with other units (i.e., carried on top the boxes) which is contingent on forming and coordinating the corresponding memory units (pairwise coordinations, i. e, stick1- fruit, stick2- fruit, box1- fruit, etc. might never amount to a solution). Ability to temporarily decouple mental operations from their motor-sensory expressions and to combine coordinated packets into stable functional units amenable to further coordination (that is, the ability to think and understand) separates humans from other species. Piaget articulated these notions convincingly, by pointing at the “contrast between step-by-step material coordinations and co-instantaneous mental coordinations” and demonstrating in multiple experiments that “mental co-ordinations succeed in combining all the multifarious data and successive data into an overall, simultaneous picture which vastly multiplies their power of spatio-temporal extensions…” (Piaget, 1978, p. 218).

### Step 4. Architecture for Understanding

**FIGURE 9:**
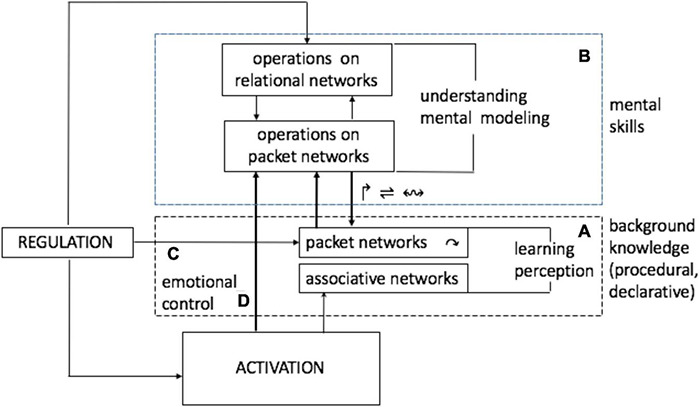
Architectures for understanding. This diagram represents cognition as a regulatory process that is directed at adapting (matching) behavior variations in the organism to condition variations in the world stream and is powered by energy inflows extracted from the stream. Organization in the system comprises different structures submitted to regulation [from tuning receptive fields in individual neurons (Fritz et al., 2003, 2005), to rotating packet vectors, to constructing and manipulating mental models], seeking to stabilize energy inflows while minimizing metabolic costs.

Figure 9 positions mechanisms of packet manipulation in the three-partite brain architecture in Figure 2 superposed on the schema of Situational Understanding in Figure 3.

Two blocks are identified, denoting two classes of memory processes and operations: block A is shared across many species while block B is exclusively human, as follows. Block A limits memory processes to the formation of associative networks and packets, and allows for the rotation of packet vectors. Block B allows other operations leading to construction of mental models and operations on them.

Block A (block B is absent or underdeveloped) reflects cognitive capacities in non-humans, from simple organisms to advanced animals. Rudimentary forms of learning reduced to selective formation and strengthening of associative links are available in simple organisms (e.g., worms, frogs) and decorticated animals [e.g., rats having 99.8% of the cortex surgically removed (Oakley, 1981)]. Intact rodents occupy an intermediate position in the capacity ladder [learning involves formation of a few neuronal groups that get selectively re-combined depending on changes in the situation (Lin et al., 2006)]. Apes and some avians can learn to coordinate a few objects (link C).

Block A operates on the associative and packet network in block B while leaving the mosaic of associative links intact. Flexible neuronal “maneuvers” [fluid intelligence (Cattell, 1971, 1978)] underlie management of competing goals and other executive functions (Mansouri et al., 2009, 2017) and involve selective re-combination of packets, producing a hierarchy of relational models (hierarchy of flexible relational structures developing on top of an associative network is called *virtual associative network).* Interactions between levels are two-directional, with the top-down processes selectively engaging lower levels, down to deployment of sensorimotor resources which can entail changes in the bottom associative network due to sensorimotor feedback (please see below).

**FIGURE 10:**
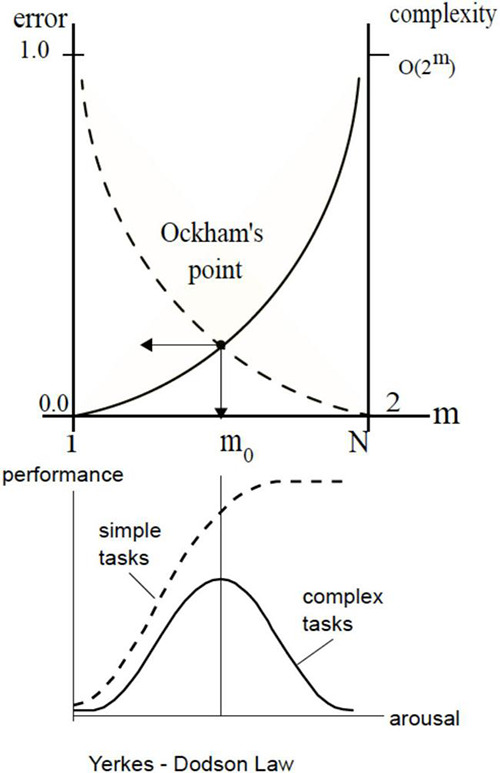
The shape of the energy landscape is a function of interplay between arousal and value distribution across the packets (reflecting value distribution in the corresponding objects). Heightened arousal lowers energy barriers across the landscape enabling coordination of distant packets, as might be necessary for unfamiliar and complex (creative) tasks, while decreasing arousal elevates the barriers thus restricting coordination to proximal packets (which might suffice for simple and familiar tasks).

Link D places energy distribution across the packet network under regulatory influence (volitional control), thus making it an integral part of a human cognitive system, as suggested in Figure 10.

In the extreme, low barriers allow floods of irrelevant associations while high barriers confine attention to a few familiar associations. Accordingly, optimal arousal obtains optimal task space partitioning (*m_0*) yielding optimal performance.

Arousal-induced changes in the landscape account for the levels of awareness, from vegetative wakefulness (flat landscape) to understanding-based awareness (optimal landscape, see Figure 1). Subjective experience of arousal varies from fear, stress, anxiety on the one and of the spectrum to excitement and exhilaration on the other end. Accordingly, moving along the spectrum changes the topological characteristics of the energy landscape: from fragmented access (i.e., some areas are inaccessible) to the unrestricted accessibility of a flat surface. Stress-induced changes in landscape topology are likely to underlie the idea of “suppressed memories” treated in psychoanalysis (disturbing memories are not erased or degraded but become “walled off” behind high barriers, so access to them can be restored if the barriers are lowered. Treatment that concentrates on the associative neighborhood (see Figure 6), as in dream analysis, seems to be appropriate for that purpose). Methods of memory recovery were disputed, on the grounds that it might be as likely to conjure false memories as to recover access to the lost ones (Loftus and Ketcham, 1996). However, creation and suppression are two sides of the same coin, i.e., the same mechanism that facilitates creative re-combination of memory structures can block access to some of them. Stress-induced landscape distortions can be responsible for other psychological symptoms, such as obsessive thoughts.

We will now return to the examples above, this time applying the notions of the VAN framework. The USS Stark incident and the Maginot catastrophe were not a product of insufficient training or illogical reasoning but resulted from understanding failure, that is, the inability to form “mental co-ordinations … combining all the multifarious data and successive data into an overall, simultaneous picture” (Piaget, 1978, p. 218). Despite differences in circumstances, the nature of cognitive deficiency was the same in both scenarios: an inability to overcome the resistance of elevated energy barriers, which resulted in fragmented (as opposed to simultaneous) “pictures.” On the VAN theory account, the captain’s mental model in the first scenario comprised two uncoordinated packets: *A* = ship, objects relevant to the ship, and *B* = all other objects. A highly valued but erroneous AWAC classification placed the Iraq jet in the second group, and the captain’s mental skills did not allow crossing the A | B barrier and coordinating members of B with members of A. In the second scenario, mental model of the high command comprised *A* = fortifications, defended area in front of fortifications and *B* = adjacent areas, objects in the adjacent areas separated by an energy barrier that turned out to be insurmountable due to overvalued significance of past experiences. French high command, as a collective entity, demonstrated low level of self-control under fear and anxiety brought about by the anticipated German attack, which caused them to fall back on the past tactics and made them “fanatically uninterested” in deviating from them.

**FIGURE 11:**
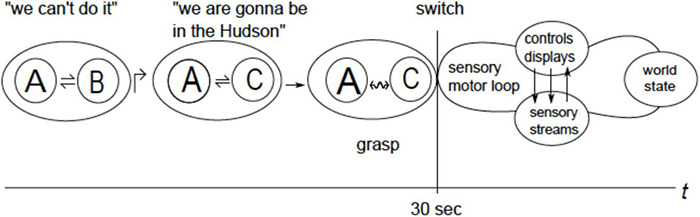
Here A - aircraft, B - New Jersey airport, C - Hudson River. Mental operations are accompanied by imagery and remain decoupled from the motor-sensory feedback until, following grasp, the motor-sensory system gets engaged.

By contrast, a high degree of self control (“ability to operate effectively under pressure, self-control in hazardous situations” Suhir, 2013) demonstrated in the Airbus incident made possible suppressing fear and bringing arousal to a level enabling situational understanding manifested in overcoming the inertia of training and customary practices (regulations, authority of the ground control, etc.), “feeling” the appropriate course of action, and making decisions at a substantive level (“we can’t do it,” “we’re gonna be in the Hudson”). The well coordinated mental model regulated subsequent activities in a top-down fashion, by selectively engaging skills and knowledge in the pilot’s background repertoire as necessary for coordinating flight pattern with river characteristics to enable a safe ditching. Figure 11 depicts a succession of mental operations.

Figure 10 underscores that mental models are regulatory structures that, beside supplying “pictures,” control their own execution *via* dynamic coordination of various data streams in the motor-sensory loop completed *via* environmental feedback [sensory streams include visual input (e.g., river shape), motor-kinesthetic input, etc.]. Execution is accompanied by a feeling of confidence in reaching the objective (e.g., safe ditching) that varies depending on the varying degree of correspondence between the envisioned outcomes of control actions and the actually observed ones. Technically, grasp can be said to establish a functional on the space of packet vectors that returns confidence values for different patterns of inter-packet coordination. Behavior of the functional depends on the vector space topology, i.e., accessibility between packets.

Following grasp, the repetitive successful exercise of a newly formed model causes its stabilization, which is captured, to an extent, in the concept of frame (schema, script, etc.) defined as a fixed memory arrangement comprised of components (slots) with variable contents (e.g., script of visiting a restaurant comprises slots “entering,” “being seated,” “studying menu,” etc. (Schank and Abelson, 1977; Norman, 1988). A few comments on the frame idea are offered in the discussion part.

Since the VAN theory pivots on the notion of energy efficiency in the brain, a brief excursion into that subject is in order. The notion that neuronal system optimizes energy processes (Yufik, 1998, 2013; Yufik and Sheridan, 2002) is consistent with later theoretical proposals (e.g., Niven and Laughlin, 2008; Vergara et al., 2019; Pepperell, 2020, 2018) and an increasing number of experimental findings (the discussion section offers a brief review of some data). To appreciate the sources of energy efficiency inherent in the VAN concept, consider the following. In an associative network, excitation in any node or group of nodes can propagate throughout the entire network. By contrast, propagation of excitations induced within a packet is obstructed by boundary energy barriers (i.e., crossing a barrier incurs energy costs). Moreover, seeking further energy savings drives the system toward constraining intra-packet activities to packet subsets and, when crossing the barriers, to engage only packets amenable to mutual coordination. In this way, formation of mental models comprising entities (packets), behavior (transition between intra-packet activity patterns) and relations (inter-packet coordination) expresses the dual tendency to increase the efficacy of action plans (enabled by situation understanding) while decreasing the costs of such planning. A reference to neuronal processes that might be responsible for some of these phenomena will conclude this section.

Interaction between neuronal cells is mediated by several types of substances, including neurotransmitters and neuromodulators. Neurotransmitters act strictly locally, i.e., they are released by a pre-synaptic neuron and facilitate (or inhibit) generation of action potentials in a single post-synaptic target. By contrast, neuromodulators act diffusely, i.e., they are released to a neighborhood as opposed to a specific synapse and affect a population of neurons in that neighborhood possessing a particular receptor type (metabotropic receptors). Neuromodulators control the number of neurotransmitters synthesized and released by the neurons, thus allowing up- or down- regulation of interaction intensity. Neurotransmitters move through fast-acting receptors metabotropic receptors are slow-acting receptors that modulate the functioning of the neuron over longer periods (Avery and Krichmar, 2017; Pedrosa and Clopath, 2017). Neuromodulators were found to provide emotional content to sensory inputs, such as feelings of risk, reward, novelty, effort and, perhaps, other feelings in the arousal spectrum (Nadim and Bucher, 2014). It can be suggested that James’ vivid depiction of “hovering of the attention in the neighborhood of the desired object” provides an accurate introspective account of the work invested in regulating neuromodulator concentration and neurotransmitter production at the packet boundary, which amounts to lowering the energy barrier until “the combined tensions of neural processes break through the bar” (James, 1950/1890, v. 1, p. 586). Since neuromodulators are slow acting, the packet remains accessible for a period of time sufficient for the task at hand.

To summarize, psychology usually treats awareness as a necessary but insufficient prerequisite for reaching understanding (e.g., one can be fully aware of all the pieces and their positions on the chessboard but fails to understand the situation). According to the present theory, predicating situation awareness on situation understanding, as in Figure 1, refers to *understanding-based awareness* (see section “Levels of Awareness”) and expresses a keen insight consistent with one of the key assertions in the VAN theory: the experience of attaining understanding accompanies emergence of a synergistic (coherent and cohesive) mental models, simulating (envisioning) possible actions on particular elements in such models generates awareness of the constraints and likely consequences of those actions in the other elements throughout the model (hence, the *situation awareness*).

## Integrating Virtual Associative Network Into the Variational Free Energy Minimization Framework

The Free Energy Minimization principle offers a “rough guide to the brain” (Friston, 2009) and extends to any biological system, from single-cell organisms to social networks (Friston, 2010). The central tenets of the VFEM come from the realization that any living system must resist tendencies to disorder, including those emanating from the environment, while obtaining means for resistance from that same environment:

“The motivation for the free-energy principle … rests upon the fact that self-organizing biological agents resist a tendency to disorder and therefore minimize the entropy of their sensory states” (Friston, 2010, p. 293).

The success or failure of the enterprise depend on the system’s ability to adapt, *via* forming models of the world used to predict the forthcoming conditions. The VFEM principle expresses this insight in information-theoretic terms, *via* the notion of variational free energy defined as follows:

“Free-energy is an information theory quantity that bounds the evidence for a model of data … Here, the data are sensory inputs and the model is encoded by the brain. ….. In fact, under simplifying assumptions…it is just the amount of prediction error” (Friston, 2010, p. 293).

Technically, variational free energy is *F*_*v*_isdefined as surprise (or self-information) - ln p (y| m) associated with observation y under model m, plus the difference between the expected and the actual observations (i.e., the prediction error under model *m*), measured as a Kullback-Leibler divergence *D*_*KL*_, or entropy, quantifying distinguishability of two probability distributions.

This section adopts the simplifying assumptions and equates variational free energy to prediction error. The VFEM principle conceptualizes minimization of prediction error as a causal factor guiding interaction with the environment, as follows:

“We are open systems in exchange with the environment; the environment acts on us to produce sensory impressions and we act on the environment to change its states. This exchange rests upon sensory and effector organs (like photoreceptors and oculomotor muscles). If we change the environment or our relationship to it, sensory input changes. Therefore, action can reduce free-energy (i.e., prediction errors) by changing sensory input, whereas perception reduces free-energy by changing predictions” (Friston, 2010, p. 295).

Adaptive capacities culminate in the ability to adjust accuracy, or precision to optimally match the amplitude of prediction errors, as follows:

“Conceptually, precision is a key determinant of free energy minimization and the enabling – or activation – of prediction errors. In other words, *precision determines which prediction errors are selected* and, ultimately, how we represent the world and our actions upon it. ….it is evident that there are three ways to reduce free energy or prediction error. First, one can act to change sensations, so they match predictions (i.e., action). Second, one can change internal representations to produce a better prediction (i.e., perception). Finally, one can adjust the precision to optimally match the amplitude of prediction errors” (Solms and Friston, 2018).

The VAN theory instantiates the VFEM principle for the human brain, identifying understanding with a particular strategy for predictive error reduction and a particular form of precision adjustment. In this way, the VAN theory proposes some substantive contributions to the VFEM framework, including the following. Firstly, the VFEM principle envisions changing actions to change sensations and changing internal representations in order to change perceptions. The VAN theory envisions, in addition, changes in the internal models to produce and change understanding. Secondly, “the motivation for the free-energy principle …. rests upon the fact that self-organizing biological agents resist a tendency to disorder and therefore minimize the entropy of their sensory states” (Friston, 2010, p. 293). VAN postulates that self-directed construction of mental models constitutes a form of self-organization in the brain that reduces the entropy of its internal states (Yufik, 2013, 2019) (more on that important point in the next section). Thirdly, according to the VFEM, error minimization brings about the minimization of energy consumption in the brain. By contrast, VAN attributes ontological primacy to energy processes and derives error reduction from the pressure to reduce energy consumption.

Technically, the VAN and VFT share the same commitment to finding the right balance between accuracy and complexity, i.e., the right kind of grouping or course graining that conforms to Occam’s principle. This follows because variational free energy is a bound upon the log of marginal likelihood or model evidence (i.e., negative surprise or self information). As noted above, the marginal likelihood can always be decomposed into accuracy and complexity. This means that the energy landscapes above map gracefully to the variational free energy landscapes that attend the free energy principle. The link between the informational imperatives for minimizing prediction errors and the thermodynamic imperatives for efficient processing rest upon the complexity cost, that can be expressed in terms of a thermodynamic cost (*via* the Jarzynski equality). An example will illustrate the underlying notion of efficiency from both a statistical and thermodynamic perspective:

Consider a frog trying to catch flies and getting disappointed by the results (too many misses). To secure a better energy supply, the frog can start shooting its tongue faster, more often, etc. If the hit/miss ratio does not improve and the frog keeps shooting the tongue in vain, it will soon sense the amplitude of prediction error unambiguously – by dying from exhaustion. Presume that neuronal mechanisms emerge that improve the score by improving sensory- motor coordination. In principle, this line of improvement could continue indefinitely making the frog progressively more sophisticated hunter, except that the mechanisms can require more neurons engaged in more intense activities which will result in increasing energy demands that can outweigh increases in the intake (besides, there are obvious physiological and physical limitations on the brain size, and neither neurons can become smaller, nor the underlying chemical processes can run faster).

**FIGURE 12:**
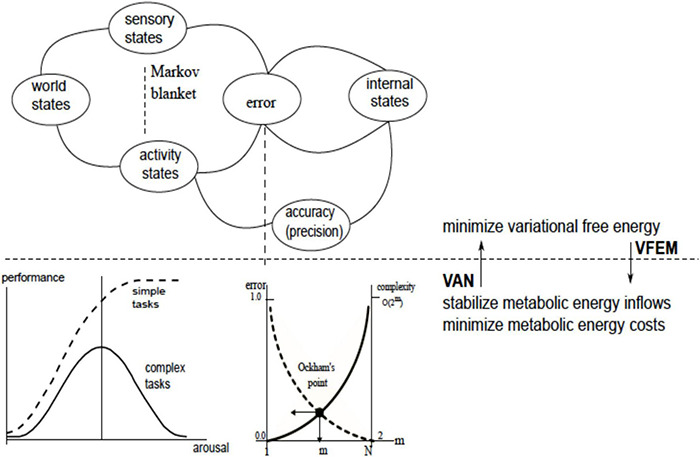
VAN theory instantiates VFEM framework, by accounting for error optimization mechanisms underlying human understanding. The figure above the horizontal dotted line depicts a VFEM construct where adaptive interaction between world states and the brain (internal states) is conducted *via* motor-sensory loop and pivots on the mechanisms of error minimization and precision adjustment (adopted from Solms and Friston, 2018). Figure below the horizontal dotted line summarizes the VAN approach deriving error optimization from capabilities inherent in self-directed packet manipulation (see Figure 9). Vertical dotted lines suggest mapping between the VFEM and VAN constructs. The sensory and activity states constitute Markov Blanket that shields internal states from the world states and, at the same, mediates interaction between them.

Consequently, radical behavior improvements are predicated on discovering mechanisms that deliver them without increases in the size of neuronal pool and/or neuronal activities, that is, without increases in internal energy consumption or, better yet, entailing energy savings. The point is that such mechanisms might or might not emerge, and error reduction is a consequence of their development, as opposed to such mechanisms being a guaranteed accompaniment of error reduction. With these caveats, Figure 12 suggests a straightforward integration of the VAN model into the VFEM framework.

## Machine Situational Understanding

This section illustrates the function of machine situational understanding and discusses approaches toward its implementation.

### Machine Understanding

A machine can be said to possess situational understanding to the extent it can:

(a) accept task definition from the operator expressed in substantive terms,

(b) evaluate a novel, unfamiliar situation and develop a course of action consistent with the task and situational constraints (the available time, data sources, etc.) and

(c) communicate its decisions and their reasons to the operator in substantive terms.

In other words, decision aid is attributed a degree of situational understanding if the operator feels that the machine input contributes into his/her situational awareness and can be sufficiently trusted to adjust his/her own situational understanding and to act on machine advice. In the VAN framework, substantive expressions address objects (entities), their behavior, and forms of behavior co-ordination (relations). The same three examples will illustrate these suggestions.

In the USS Stark incident, an on-board situation understanding aid (SUA) could overrule AWAC target classification and issue a warning like “Attention: there is 0.92 probability that this is enemy aircraft.” The chances that the warning will be trusted and acted upon will improve significantly if, when asked “How do you know?” the system would reply with “The aircraft was ascending but then turned sharply and started descending and accelerating toward you.” Assuming that the captain interacts with the ship systems *via* the aid, the SUA would accept the captain’s command “Engage the target” and initiate activities by the engagement protocol [note that learning systems (e.g., deep learning) are capable of reliably detecting and identifying objects but are limited in their ability to apprehend relations and explain their decisions to the user].

In the Airbus incident, the SUA could be tasked with interacting with ground control to request permission to land in NJA, and could respond with “We are not going to make it.” Improving situation understanding in the Maginot scenario would require breaking a rigid mental template, some (tentative) suggestions for a possible role of SUA will be made shortly, after introducing VAN computational framework.

### Virtual Associative Network Computational Framework and Virtual Associative Network/Variational Free Energy Minimization Integration

The VAN computational framework was dubbed “gnostron” (Yufik, 2018), to underscore distinction from “perceptron”: perceptron has a fixed neuronal structure while gnostron is a neuronal pool where structure evolves gradually and remains flexible. Gnostron formalism is a straightforward expression of VAN considerations summarized in section “Integrating Virtual Associative Network into the Variational Free Energy Minimization Framework,” as follows.

World is a stream of stimuli *S* = *s_1*, *s_2*, …., *s*_*M*_ arriving in different combinations at a pool comprised of N neurons *X* = *x*_1_,*x*_2_,*x*_*N*_, with each neuron responding probabilistically to a subset of stimuli. In turn, the stimuli respond probabilistically to the neurons that pool mobilizes and “fires at” them, by releasing energy deposits (neuron*x*_*i*_ has receptive field μ_*ij*_,μ_*ih*_, …μ_*ik*_, here μ_*ih*_ denotes probability that stimulus *s_h* will release deposit *E_h* in response to the pool having fired *x_i*). Mobilization (selecting neurons and preparing them to fire) takes time and neurons, after having fired, need to time to recover, which forces the pool to engage in anticipatory mobilization. Engaging *x*_*i*_consumes energy δ_*i*_ comprising the work of mobilization ρ_*i*_ and the work of firing ν_*i*_, δ_*i*_ = ρ_*i*_ + ν_*i*_ (note that mental operations are constituents of mobilization).

**FIGURE 13:**
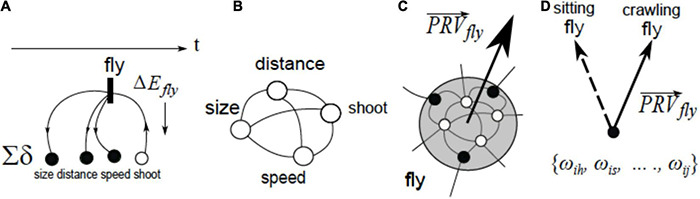
Frogs shoot their tongues at small moving objects in sufficient proximity. **(A)** Mobilizing, firing and inhibiting neurons consumes energy and extracts energy deposits from the world stream (the flies). **(B)** Connections are formed between co-firing neurons and get strengthened with each successful episode (these connections can be genetically fixed, as in frogs). **(C)** Phase transition in the network turns the associative group into a cohesive packet bounded by an energy barrier. **(D)** The packet is a functional unit (has a receptive field computed as a function of receptive fields in the constituent neurons) amenable to mobilization and allocation. In the humans, mobilizing and allocating packets is experienced as perceiving “objects,” packet “tuning” (rotating packet vector) defines the states of the object, admissible transitions between the states define behaviors (self-directed packet manipulation is available to humans but not to frogs).

The pool’s survival depends on maintaining net energy inflows (cumulative deposits minus cumulative expenditures) above some minimal threshold, which includes the requirement that the average mobilization period is commensurate with the tempo of stimuli arrival. This formulation translates the problem of survival and adaptive efficiency into that of probabilistic resource optimization: orchestrate the pool’s activities (mobilization, firing and inhibition, or demobilization) consistent with variations in the stream so that energy inflows are maximized (or stabilized at some acceptable level) while energy expenditures are minimized. Conceptualizing cognitive processes as dynamic optimization of neuronal resources (Yufik, 1996, 1998) is consistent with the recent views associating advanced cognitive functions with the ability to monitor the significance of multiple goals and flexibly switch between them so that the rewards yielded by the goals are maximized and the associated neuronal costs are minimized (e.g. Mansouri et al., 2017). The gnostron framework pivots on the notion that mechanisms of neuronal groupings envisioned in the VAN map directly onto heuristics for probabilistic resource optimization so that energy savings in the biological substrate equate to reduced processing expenditures in the machine implementation. Figure 13 returns to hunting frogs (section “Integrating Virtual Associative Network into the Variational Free Energy Minimization Framework”) in order to illustrate and summarizes these notions,

Boundary energy barriers bound evidence for the corresponding object (Yufik and Friston, 2016; Yufik, 2019, 2021a,b). More precisely, recognition confidence associated with firing a neuron is a function of the corresponding probability μ_*ih*_ in the neuron’s receptive field and the strength of neuron’s attachment to (correlation with) other neurons in the packet. High confidence motivates leaving the packet but the fee charged for crossing the barrier discourages premature decisions and forces seeking confirmation or disconfirmation, in which case paying the fee remains the only option.

Technically, formation of packets constitutes a heuristic yielding complexity reduction in the probabilistic optimization problem. More precisely, forming packet network atop the associative network breaks a very large, continuous problem into a succession of discrete problems small enough to be solved by full search (this strategy appears to underlie the Long Term Memory/Short Term Memory (STM) architecture where small STM buffer [less than 10 items (Miller, 1956)] is subject to exhaustive scanning (Sternberg, 1969). The computational architecture of associative cortices readily affords self-partitioning in associative networks allowing near-optimal behavior. In Gnostron, the partitioning quality is defined by a simple criterion: choose a particular optimization algorithm and compare results obtained before (baseline) and after partitioning into packets [a stripped down, proof-of-concept system for target recognition obtained close to two orders of magnitude complexity reduction with acceptably small error amplitude (Malhotra and Yufik, 1999; Yufik and Malhotra, 1999)]. Figure 13 generalizes the gnostron proposal.

**FIGURE 14:**
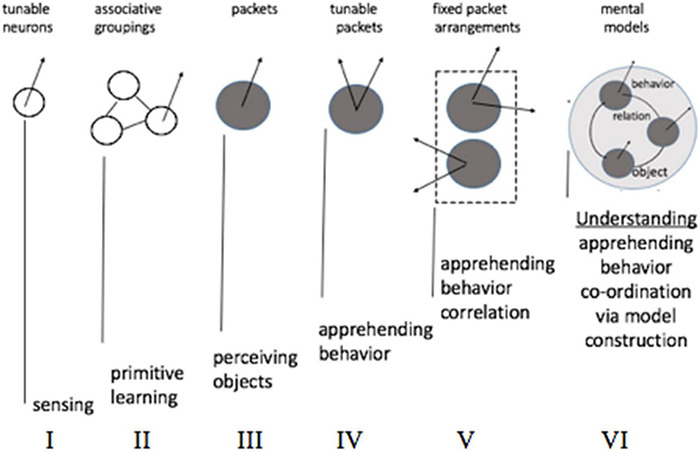
VAN identifies six basic mechanisms employed by the brain to produce adaptive responses to variations in the world stream. The mechanisms vary from tuning receptive fields in individual neurons **(I)** to constructing and manipulating mental models **(VI)**. Gnostron architecture integrates these mechanisms.

Figure 14 lists key neuronal mechanisms postulated in VAN, seeking to establish three points: First, the postulated mechanisms have algorithmic expression in the framework of probabilistic resources optimization. Second, gnostron framework establishes a degree of isomorphism between human decision processes (as envisioned in VAN) and computational procedures: both substantive decision-making and Gnostron procedures operate with models representing objects, behavior and relations. Moreover, lower level gnostron procedures can be mapped meaningfully onto mental operations (for example, computing packets involves operations on cutsets in networks that correspond, roughly, to refocusing attention from prominent relations between objects to background relations that were deemed to be less significant). Finally, gnostron mediates between human operators and other systems but does not replace them (for example, gnostron can be calling on standard on-board systems to estimate the chances of safe landing in the New Jersey Airport. By the same token, it will be able to respond to a query like “Is the NJA an option?”). In this way, gnostron shields an operator from computation details while maintaining interaction at a substantive level adequate for shared situational understanding.

Strategy V involves formation of fixed templates. To appreciate differences in performance yielded by strategies V and VI, map them onto acquisition of chess skills, as follows: strategy III enables one to tell apart (recognize) chess pieces, strategy IV associates admissible behavior (rules) with the pieces, and strategy V enables memorization of particular tactics. To take a closer look at the latter, a few chess notions will be helpful: Fool’s mate (capital F) is a checkmate delivered in the fewest possible moves (2–4) after the beginning of the game, fool’s mate (small f) is a maneuver of a few moves anytime in the game that delivers checkmate or turns opponents’ position into a hopelessly lost one, and Sicilian defense is a particular Black move in response to a particular White move at the opening of the game (1. e4 c5). It’s easy to see that a novice player taught only the Sicilian template is unlikely to seek tournaments (“what will happen after I do c5?”). Chess books teach seven basic strategies for continuing the game but, being taught all seven or, to take things to the extreme, having memorized the gazillion games ever played that used Sicilian template would make no difference: fool’s mate is guaranteed if a more skilled opponent deviates from one of the memorized games, or just opens the game by any move other than e4.

The argument is (a re-statement of Searle’s Chinese room argument) that knowledge, however, extensive, neither amounts nor guarantees understanding. Moreover, knowledge without understanding easily becomes a vulnerability. More to the point, the German army delivered fool’s mate to the French command at the beginning of the campaign, taking advantage of the fact that the latter adhered to a rigid tactical template acquired in the WWI. Deficiencies in strategic thinking on that scale can hardly be remedied by a decision aid (although detecting rigid templates can be a part of gnostron tactics when interacting with human operators).

**FIGURE 15:**
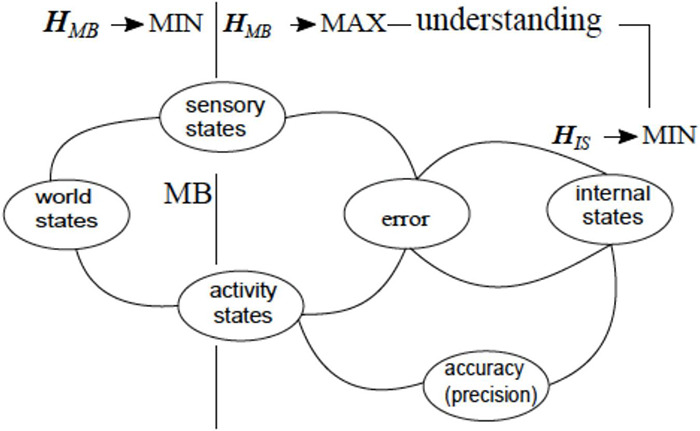
An absence of understanding capacity entails tendency to minimize the entropy in a Markov Blanket while understanding seeks to maximize entropy in MB while minimizing entropy of the internal states [entropy of associative network is maximal if potential connectivity is unrestricted (Figure 14I) and minimal when connections are restricted (by coordination demands) and sparse (Figure 14VI)].

The chess example will serve to illustrate a general contention regarding situational understanding in both the human and the machine, as follows. Understanding involves the ability to form templates that is inextricably combined with the ability to re-structure and deviate from them and to incorporate them as units into other structures. Growing understanding is accompanied by growing organization and global order in the neuronal pool (see Figure 13) and growing repertoire of sensorimotor activities (e.g., from acquiring a repertoire of standard procedures in managing routine flights to safely ditching a suddenly disabled aircraft). The expanding activity repertoire entails growing entropy in the sensory-motor system. That is, understanding capacity brings about reduction of entropy in the internal states while increasing entropy in the motor-sensory periphery. Figure 15 illustrates this important aspect of VAN/VFEM integration.

Incorporation of understanding into the VFEM schema, as in Figure 14, suggests a modification in the formulation of the principle, as follows: *F_V* → min under *H*_*MB*_ → max and *W*(*D*_*KL*_) → max.

Here, *W*(*D*_*KL*_) denotes the amount of work invested in minimizing discrepancy between the predicted and actual probability distributions [the Kullback-Leibler divergence was shown to define a lower bound to entropy production and thus the average amount of work dissipated along the process (divided by the temperature) (Roldan and Parrondo, 2012)].

That is, under a fixed energy budget in the brain, understanding capacity is a result of increased organization (decreased entropy) in the regulatory system which diverts more energy to—and thus increasing the amount of useful work in—the memory system, to allow expanding activity repertoires (growing entropy) in the motor-sensory system (see Figure 2) that in turn leads to increasing (and/or stabilizing) energy inflows extracted from the world stream. Operations on models underlie prediction and retrodiction: in A and B under relation r, changes in the behavior of A predict changes in the behavior of B and changes in the behavior B retrodict to changes in the behavior in A, as afforded by the relation r. The process is tightly constrained in a template (e.g., under conviction that frontal assault is the only viable strategy, any intelligence is interpreted as either conforming, or irrelevant, or a product of deliberate misinformation). Transition from template-matching to mental modeling relaxes the constraints, posing the problem of hypotheses selection (“that does not look like preparations for a frontal assault, what can that possibly be?”) captured in the notion of abductive inference.

“The first starting of a hypothesis and the entertaining of it, either as simple interrogation or with any degree of confidence, is an inferential step which I propose to call abduction (or retrodiction). This will include a preference for any hypothesis over others which would equally explain the facts, so long as as this preference is not based on upon any previous knowledge bearing upon the truth of the hypotheses, nor on any testing of any of the hypotheses, after having admitted them on probation. … the whole question of what one of the number of possible hypotheses ought to be entertained becomes purely a question of economy” (Peirce, 1901/1955, pp. 151, 154).

The thinking process naturally selects the path of least resistance (i.e., strong associations, as in a template), and needs to be forcefully interrupted and re-directed to paths deviating from “any previous knowledge.” These operations are defined as “intervention” and insertion of “counterfactuals” in a recent probabilistic model of causal reasoning (Pearl and Mackenzie, 2018), and are represented by operations of jump, coordination and blending in VAN (to be discussed elsewhere).

To summarize, this section mapped some of the cognitive operations claimed to underlie understanding capacity in the humans onto computational procedures defined within the probabilistic optimization framework [excepting some residue having no computational expression (Penrose, 1997)]. It was proposed that understanding allows the brain to deal with non-contiguous, weakly correlated stimuli groupings in the world stream. In particular, understanding makes possible accounting for complex interdependencies between actions and world states, as in producing changes in objects indirectly, *via* coordinated changes in some other objects. Cognitive operations boil down to variable grouping and stabilization of the groups which enables subsequent intra-group variation and inter-group coordination, all serving to maximize and stabilize energy rewards (value) while minimizing internal energy costs. These operations can be mapped onto brain components whose functions have been defined in classical models as well as in some recent findings [e.g., the hippocampus has been found to be constructing abstract values spaces (Knudsen and Wallis, 2021)]. Emphasizing the role of coordination in understanding is consistent with a classical theory (Piaget, 1975, 1978) and with some recent findings concerning the role of cerebellum in the higher cognitive functions (Cerminara et al., 2009; Schmahmann et al., 2019).

Implementing operations postulated in the cognitive theory in tractable algorithms would endow machines with capabilities approximating those attributed to human situational understanding within selected situation classes. In particular, machines could be approaching the ability to “feel” the direction of appropriate actions without examining details and to formulate recommendations, explain them and receive instructions from human operators expressed in substantive terms. Attaining situational understanding reduces operational complexity (Yufik and Hartzell, 1989) enables explainable predictions, identification of critical situational elements and dynamic orchestration and optimization of cognitive and computing resources (Lieder and Griffiths, 2019).

## Discussion

Arguably, foundational ideas of the cognitivist framework were influenced by von Neumann’s conceptualization of computing systems envisioning that data and procedures for operating on the data are held in the same medium. The template “data – procedures” holds no “slots” for understanding so adopting the template in representing cognition required marginalizing the role of that capacity in intelligent performance. Accordingly, a definitive volume on human problem-solving mentioned understanding once in the concluding chapters, and only to point out that “high level of mechanization can be achieved in executing the algorithm, without any evidence of understanding” (Newell and Simon, 1972, p. 832). The cognitivist framework accorded understanding no function in the architecture of cognition (Anderson, 1983; Rosenbloom et al., 1991] nor any place in a theory of cognition (Newell, 1992), and structured the definition of understanding so it could be forced into the available two “slots”:

“S understands knowledge K if S uses K whenever appropriate. S understands task T if S has knowledge and procedures needed to perform T” (Simon, 1979, p. 447).

**FIGURE 16:**
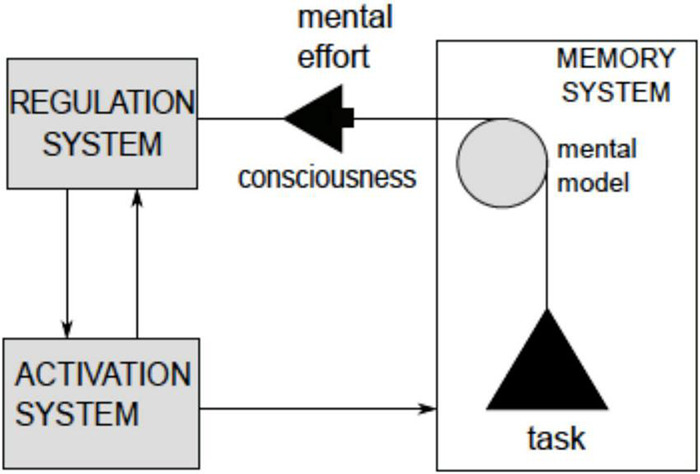
Adapting to world streams involves work performed by regulatory system on a memory system. Mental models are neuronal structures amplifying the brain’s capacity to handle challenging tasks (non-contiguous stimuli, acting on objects to impact other objects, etc.) within a limited energy budget (Raichle and Gusnard, 2002) (question “who is pulling the rope in the pulley?” will be entertained elsewhere).

Language understanding was conceptualized as manipulation of scripts (i.e., template matching) (Schank and Abelson, 1977). It is interesting to note that a book addressing the practice of problem solving as opposed to the theory of that in Newell and Simon (1972), presented in the front-page picture some key notions that were overlooked in the theory: the brain was depicted as a contraption comprising a power plant, a regulator and a system of wheels delivering power to a pulley used for lifting weights (Fogler et al., 2013). Figure 16 borrows from that depiction to re-state a main message of this paper.

Conceptualizing cognition as mental work invested in dynamic orchestration and marshaling of neuronal resources suggests a simple definition of consciousness, as follows (we are taking the liberty of citing an earlier work):

“Virtual networks form spontaneously on top of the associative network. By contrast, operations on the virtual network are not spontaneous but self-directed (deliberate, attentive, conscious) and are conducted by the control module. These operations perform work and require cognitive effort, the term “consciousness” denotes the experience of exerting that effort. On that account, “cogito ergo sum” expresses not an inference but a direct experience of cognitive strain – one can doubt the reality of the objects of thinking and even of the subject of thinking but cannot doubt the immediate and direct experience of an effort exerted in the process of thinking” (Yufik, 2013, p. 50).

In short, VAN suggested that cognitive processes alternate between conscious (deliberate, effortful) and subconscious (spontaneous) phases. It is encouraging that later studies have arrived at similar conclusion in treating the phenomenon of consciousness (Solms, 2021).

In general, the VAN approach allowed drawing a line from neuronal processes all the way up to understanding and consciousness. The line is admittedly thin and punctuated but short (only 4 waypoints), connecting basic experimental findings [“tunable” neurons (Fritz et al., 2003, 2005), “tunable” assemblies (Georgopoulos and Massey, 1987; Georgopoulos et al., 1989, 1993)] to most advanced cognitive theories (Friston and Stephan, 2007; Friston, 2009, 2010; Parr and Friston, 2019; Ramstead et al., 2021). The approach builds on some of the key insights at the foundation of cognitive science [neuronal assemblies (Hebb, 1949, 1980), fluid intelligence (Cattell, 1971, 1978), mental effort in memory retrieval (James, 1950/1890), understanding as co-instantaneous co-ordination (Piaget, 1978, 1975), mental modeling (Johnson-Laird, 1983), other], and anticipated some of the recent ideas and suggestions relating energy processes and cognition (Christie and Schrater, 2015; Pepperell, 2018; Vergara et al., 2019; Hylton, 2020). Consistent with the recent analysis of different kinds of free energy in the Bayesian account of cognition (Gottwald and Braun, 2020), the VAN model establishes reciprocity between the minimization of variational free energy and minimization of thermodynamic free energy in the neuronal system (Yufik and Friston, 2016; Yufik et al., 2016). Tentatively, the approach suggested unity of or a close relation between the mechanisms of sensori-motor coordination (Sparrow and Irizarry-Lopez, 1987; Sparrow and Newell, 1998; Sparrow et al., 2007; Latash, 2008, 2021), cortical coordination (Bressler and Kelso, 2001) and coordination in mental models (Yufik and Friston, 2016; Yufik, 2019, 2021a,b). Finally, the approach informs design of operator support in complex dynamics tasks (Yufik and Hartzell, 1989; Yufik and Sheridan, 1997; Landry et al., 2001; Yufik and Sheridan, 2002) using a transparent mathematical formalism (Yufik, 1998). Arguably, the hierarchy of VAN processing mechanisms (as in Figure 13) is compatible with the idea of “neuron-centered concepts” that associates concepts with patterns of input information evoking specific selective responses in groups of neurons (Gorban et al., 2019) [VAN postulates existence of complex neurons responding to specific activity patterns in lower-level (simpler) neurons or neuronal groupings]. The VAN view is consistent with the notion of cognition grounded in modal simulations, bodily states, and situated actions (Barsalou, 2008), as opposed to more conventional view in AI reducing cognition to computations on amodal symbols. As was argued earlier in this section, the conventional (cognitivist) approach has been downplaying the role of understanding in intelligent performance.

With some exaggeration, the view on cognition adopted in AI and cognitive science can be characterized as “intelligence without understanding.” Figuratively, human intelligence can be compared to an Egyptian pyramid visited by tourists who are paying attention to a few stones at the bottom (learning) and the last stone on top (reasoning) while ignoring the rest. The pyramid holds a great promise since even limited explorations have produced spectacular successes. In the period of about 60 years, during which neural network technology has progressed from handling simple tasks (like recognizing letters) to participating in the most complex form of scientific analysis (Krasnopolsky, 2013) and beating humans in the games of chess and Go. The technology is based on algebraic methods of iterative error reduction (training) which are highly computationally intense. Accordingly, the progress was due to increases in hardware efficiency and the development of ingenious heuristics aimed at reducing the computational complexity of the iteration procedures. The hardware efficiency has increased about a billion times [NVIDIA’s GTX 1080 GPU delivers nine teraflops for about $ 500, a similar power output in 1961 would have cost about $9 trillion for a string of IBM 1620 computers (Shepard et al., 2018)]. For argument’s sake, assume that the efficiency of the procedures has increased a thousand times, yielding a trillion times increase in the overall efficiency. Consider the following: the analysis of eye movements showed that expert chess players immediately and exclusively focused on the relevant aspects in the chess task while novices also examined irrelevant aspects (Bilalić et al., 2010). The ability to “feel” the situation, or to “know what should happen in given circumstances” prior to examining those circumstances in detail [(Feynman, c/f de Regt, 2017, p. 102] makes possible competition between slow thinking human players and fast computing chess machines.

The point is that the brain cannot accelerate either the underlying biophysical processes or the conscious reasoning, can neither miniaturize neurons nor increase their number, and cannot significantly increase the average rate of ATP production. These limitations foreclosed the paths to cognitive performance improvements taken in AI and enforced development of radically different strategies. A fair competition between human players and chess algorithms would require running the algorithms on an abacus or some manual calculator.

AI is being widely perceived as a critical and, perhaps, decisive component in the national defense (West and Allen, 2020; Niotto, 2021), giving an advantage that derives predominantly from the strength of machine learning in general and neural nets in particular. The expectation seems to be that friendly neural nets will be victorious over the adversarial ones, which calls for designing methods to deceiving adversarial nets (e.g., Nguyen et al., 2015) while ruggedizing own nets and preparing them for frontal assaults. Conclusion of an expert group tasked with assessing the implementation of AI for the Department of Defense appear to be curbing the expectation:

“the sheer magnitude, millions of billions of parameters (or weights) which are learned as part of the training… makes it impossible to really understand exactly how the machine does what it does. Thus the response of the network to all possible inputs is unknowable” (Scharre, 2018, p. 186).

It is interesting to note that recent developments in the neural net technology have taken a turn suggesting possible convergence with some of the methods outlined in this paper. In particular, clusters of neurons (called “capsules”) are being identified in neural nets whose activity vector is taken to constitute the instantiation parameters of a specific type of entity such as an object or an object part. With that, the length of the activity vector is taken to represent the probability that the entity exists and its orientation to represent the instantiation parameters (Sabour et al., 2017).

To main points in this paper can be summarized as follows:

1. The paper presented a definition of understanding that is consistent with and substantiating analysis of understanding capacity in the current literature (Piaget, 1978; de Regt, 2017), outlined several hypotheses concerning the underlying mechanisms (the VAN theory) and suggested that (a) understanding constitutes a special form of Active Inference and (b) situational understanding enables situation awareness, consistent with the conceptualization expressed in Figure 1.
2. The active inference framework encompasses the entire spectrum of living organisms and associates adaptive behavior with the minimization of variational free energy in the nervous system (Friston, 2010). According to VAN, understanding engages mechanisms that are unique to humans and yield a dual benefit of decreasing both the variational free energy and the metabolic energy expenditures. Minimization of variational free energy roughly equates to minimizing prediction error. Prediction *via* understanding provides a uniquely efficient form of error reduction.
3. The notion that minimization of metabolic costs can serve as a unifying principle in considering brain processes is not new (e.g., Hasenstaub et al., 2010; Huang et al., 2012). The VAN proposal deviates from the other suggestions, by (a) identifying specific mechanisms of metabolic cost minimization and (b) associating these mechanisms with a potentially unlimited growth in the variety and complexity of tasks accessible to humans, including the ability to overcome the inertia of past learning and to act efficiently under fluid and novel circumstances having no past precedents (Yufik and Sheridan, 2002; Yufik, 2013).
4. Understanding involves self-directed composition of coordinated neuronal structures (mental models) establishing relations (dependencies) between entities perceived previously as separate and independent. Composing such models can be highly effort-demanding. However, such composition expenditures are compensated by low-effort manipulations of the models making one aware of how local changes can bring about and coordinate with changes in the rest of the model (e.g., Yufik and Yufik, 2018). More precisely, manipulating models can “give some feel for the character” of coordinated changes (de Regt, 2017), which subsequently focuses attention on the critical situation elements. In general, mental modeling enables advances in the performance of complex tasks, by minimizing both the internal costs of the foresight and the risk of costly errors.
5. The paper used the notion of binary neurons, but only to simplify the argument. The theory is not restricted to this simplification, hypothesizing the existence of classes of complex neurons responding to different activity patterns in their input, to combinations of such activity patterns in several neuronal groups, or to forms of activity coordination [e.g., “concept cells” responding to different images of a person as well as the written and spoken names of that person (Quiroga, 2020) belong to the second class]. The pivotal notion of packets defines a property of neuronal groups that is invariant across models of neurons [in the same way as the notion of “neuronal assembly” (Hebb, 1980) is not committed to any particular model]. The theory builds on two experimentally established and model-invariant characteristics of neuronal mechanisms [rotation of assembly vectors (Georgopoulos et al., 1989) and task-related plasticity of neuronal receptive fields (Fritz et al., 2003)], expanding their application to complex neurons and neuronal groupings.
6. The theory derives understanding from coordination in the behavior (patterns of excitation-inhibition activities) of neuronal packets, which is consistent with conceptualizing brain as a dynamical system or “dynome” [as opposed to static “connectome” (Kopell et al., 2014)]. By definition, virtual network comprises a hierarchy of network types [synaptic, associative, packet, behavioral and relational networks (Yufik, 1998, 2019)]. Roughly, the former two network types belong to neural and functional connectomes while the latter three types form a dynome. Recent literature associates advanced cognitive capabilities in primates and humans with the ability to monitor the significance of multiple goals in parallel, and to switch between the goals (Mansouri et al., 2009; Mansouri et al., 2017). The present proposal expands the scope of advanced capabilities in the humans, to include dynamic coordination of multiple goals within integrated situation models.
7. The paper argues that increasing the efficiency of human-machine systems, particularly in challenging circumstances (short decision cycle, high cost of errors, etc.) requires mutual understanding between the parties. The VAN theory suggests an avenue toward meeting the requirement, offering tractable procedures amenable to integration with the methods of active inference. The VAN formalism (gnostron) is orthogonal to methods rooted in the perceptron architecture (vector movement coordination in dynamically composed networks in the gnostron vs. vector mapping in fixed networks with adjustable synaptic weights in the perceptron).
8. Mental modeling constitutes a form of self-organization in the brain. Biological processes underlying such self-organization can be approximated computationally in conventional (von Neumann-Turing) computers or, potentially, emulated in devices operating on principles different from those adopted in the conventional machines (Hylton, 2020).
9. In machine understanding, as conceptualized in VAN, machine processes and human cognitive processes are isomorphic, i.e., humans think of entities, behavior and relations and machines compute the same. Shared situational understanding in a human-machine system does not make the system infallible but can be expected to amplify and accelerate human grasp, increase human trust and confidence, and sharply reduce the likelihood of costly errors. In the autonomous scenarios, understanding expands the range of tasks that can be reliably delegated to the machine (methods for measuring performance improvements resulting from machine understanding are beyond the scope of this paper).

The above points suggest directions for further R&D, from developing deeper insights into the role and mechanisms of understanding to formulating tractable computational formalisms and designing artifacts that take advantage of those insights. The VAN/VFEM proposal contends that the objective of ensuring battlespace dominance brings to the fore the problem of situation understanding enabling coordination and prediction of multiple activities under conditions that might be unfamiliar and undergoing kaleidoscopic changes. The proposal complements advances in machine learning and suggests other approaches that might be worth exploring.

It feels appropriate to conclude the discussion with a quote from a philosopher of mind and Nobel Laureate in physics:

“…it seems to me that intelligence is something which requires understanding. To use the term intelligence in a context in which we deny that any understanding is present seems to me unreasonable. Likewise, understanding without any awareness is

also a bit of a non-sense. … So that means that intelligence requires awareness. Although I am not defining any of these terms, it seems to me to be reasonable to insist upon these relations between them” (Penrose et al., 2000, p. 100).

## Data Availability Statement

The original contributions presented in the study are included in the article/supplementary material, further inquiries can be directed to the corresponding author.

## Author Contributions

YY contributed theoretical discussion and wrote the manuscript. RM contributed to the discussion and analyzed examples and applications.

## Conflict of Interest

YY was employed by the company Virtual Structures Research, Inc. The remaining author declares that the research was conducted in the absence of any commercial or financial relationships that could be construed as a potential conflict of interest.

## Publisher’s Note

All claims expressed in this article are solely those of the authors and do not necessarily represent those of their affiliated organizations, or those of the publisher, the editors and the reviewers. Any product that may be evaluated in this article, or claim that may be made by its manufacturer, is not guaranteed or endorsed by the publisher.
